# The soluble amino-terminal region of HVEM mediates efficient herpes simplex virus type 1 infection of gD receptor-negative cells

**DOI:** 10.1186/1743-422X-9-15

**Published:** 2012-01-13

**Authors:** Hyunjung Baek, Jae Hong Kim, Yoon Tae Noh, Heechung Kwon

**Affiliations:** 1 Division of Radiation Oncology, Korea Institute of Radiological and Medical Sciences, 215-4, Gongneung-Dong, Nowon-Ku, Seoul 139-706, South Korea; 2 School of Life Sciences and Biotechnology, Korea University, Seoul, South Korea

**Keywords:** HSV-1, HVEM/HveA, gD, Soluble entry receptor

## Abstract

**Background:**

Previous studies from our own and other labs reported the surprising finding that the soluble V domain of the herpes simplex virus type 1 (HSV-1) entry receptor nectin-1 can both block HSV infection of receptor-bearing cells and mediate infection of receptor-deficient cells. Here we show that this property is not unique to nectin-1. We generated a pair of truncated, soluble forms of the other major HSV-1 entry receptor, herpes virus entry mediator (HVEM or HveA), and examined its effects on HSV-1 infection of receptor-deficient cells.

**Results:**

In cultures of CHO-K1 cells, sHveA_102 _comprising the two amino-terminal cysteine-rich pseudorepeats (CRPs) of HVEM enabled infection of greater than 80% of the cells at an MOI of 3, while sHveA_162 _comprising the complete ectodomain failed to mediate infection. Both sHveA_102 _and sHveA_162 _blocked infection of CHO-K1 cells stably expressing HVEM in a dose-dependent manner, indicating that both were capable of binding to viral gD. We found that sHveA_102_-mediated infection involves pH-independent endocytosis whereas HSV infection of HVEM-expressing CHO-K1 cells is known to be pH-dependent.

**Conclusions:**

Our results suggest that the C-terminal portion of the soluble HVEM ectodomain inhibits gD activation and that this effect is neutralized in the full-length form of HVEM in normal infection.

## Background

Herpes simplex virus type 1 (HSV-1) infects a broad range of mammalian cells, including epithelial cells, lymphocytes, and post-mitotic neurons. Initial HSV attachment to host cells is mediated by the binding of viral envelope glycoproteins C (gC) and gB to ubiquitous heparan sulfate moieties on the surface of cells [1-3]. While not essential [4], these interactions facilitate the binding of glycoprotein D (gD) to one or more of its cognate cell-surface receptors, nectin-1 (HveC), HVEM (HveA), and 3-*O*-sulfated heparan sulfate (3-OS HS) [5-7]. Binding to these entry receptors causes conformational changes in the gD ectodomain that signal activation of the downstream effectors of HSV entry, gB and gH/gL, the proximal mediators of membrane fusion and capsid delivery into the cytoplasm [8-12]. Recent studies have also demonstrated that PILRα (paired immunoglobulin-like type-2 receptor) and non-muscle myosin heavy chain IIA can function as HSV-1 entry co-receptors through interaction with gB [13,14].

The absolute dependence of HSV-1 infection on gD binding to a cognate receptor indicates that the tropism of the virus is determined at least in part by the distribution of gD receptors. Nectin-1 is a member of the immunoglobulin superfamily and is expressed on many cell types, including fibroblasts, epithelial cells and neurons [15]. Nectins function as intercellular adhesion molecules localized to cadherin-based adherens junctions [16]. The variable (V) domain of nectin-1 is sufficient for binding to gD and the initiation of fusion between the virus envelope and cell membranes [17]. HVEM is a member of the tumor necrosis factor receptor (TNFR) superfamily and is expressed in hematopoietic cells and lymphoid tissues such as spleen and thymus [18,19]. HVEM contains four cysteine-rich pseudorepeats (CRPs) characteristic of members of the TNFR family in its ectodomain and functions as a mediator of HSV-1 and HSV-2 entry mainly into human lymphocytes [6,19]. The natural ligands for HVEM include LIGHT, lymphotoxin alpha (Lt-α), B- and T-lymphocyte attenuator (BTLA), and CD160 [20]. The contribution of the third gD receptor, 3-OS HS, to the broad HSV-1 tropism is not as clearly defined since this glycosaminoglycan modification is not easily detectable by immunological or other methods. However, novel approaches are beginning to reveal the role of this receptor [21].

X-ray crystallography has shown that HVEM binds to a flexible hairpin at the amino terminus of gD [8]. A variety of mutations in this region, including Q27P originally identified in KOS-rid1 virus [22], abolish HVEM binding [23,24]. Using a series of truncated forms of HVEM, Whitbeck and colleagues demonstrated that the two N-terminal CRPs of HVEM are required and sufficient for binding to HSV-1 gD [25]. In their study, HveA(120 t), consisting of the first and second CRP, showed full gD binding activity by competition ELISA and blocked HSV entry into CHO cells expressing HVEM.

We previously reported that the V-domain of nectin-1 as a soluble form can mediate efficient virus entry into HSV-resistant CHO-K1 cells that lack gD receptors [26]. Here, we have investigated whether soluble forms of the HVEM ectodomain have a similar ability. To this end, we constructed soluble recombinant proteins consisting of the first two CRPs (sHA_102_) or the full ectodomain (sHA_162_) of HVEM. While both soluble proteins inhibited HSV infection of HVEM-expressing CHO-K1 cells, only sHA_102 _mediated virus entry into receptor-negative CHO-K1 cells. Infection mediated by sHA_102 _was highly efficient, involved specific binding of sHA_102 _to viral gD, required heparin-sensitive virus attachment to the cells, and took place by a pH-independent endocytic mechanism as opposed to the pH-dependent mechanism mediating HSV infection of HVEM-expressing CHO cells [27]. Our results indicate that the C-terminal portion of the HVEM ectodomain contains sequences that inhibit gD activation and suggest a context effect allowing gD activation by full-length HVEM, but not the soluble ectodomain, in the acidic milieu of endosomes.

## Results

### Production of soluble gD receptors

**Figure 1:**
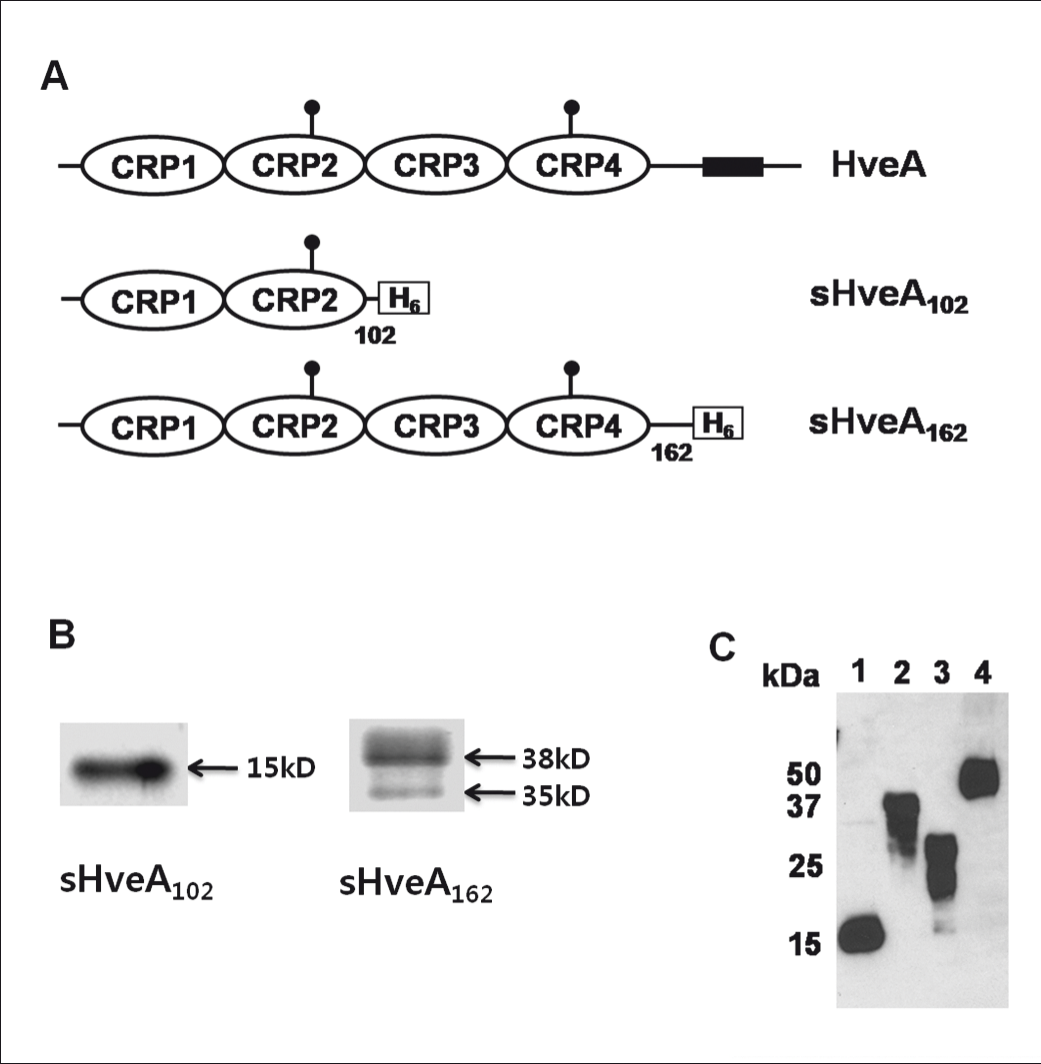
**Structure and expression of soluble HVEM proteins**. (A) Schematic representations of full-length HVEM (HveA) and the truncated HVEM proteins produced for this study. Lollipops indicate glycosylation sites and numbers indicate amino acid positions relative to position 1 of mature HVEM. CRP, cysteine-rich pseudorepeat; H_6_, six-histidine tag. (B) Silver-stained SDS-PAGE gels of soluble protein samples. (C) Western blot detection of purified recombinant soluble proteins using anti-His tag antibody. Lane 1, sHA_102_; lane 2, sHA_162_; lane 3, sNec1_123 _used in Figure 2D; lane 4, sgD_287 _used in Figure 3.

We constructed expression plasmids for two His_6_-tagged soluble HVEM proteins, sHA_102 _containing the gD-binding first two CRPs (102 aa), and sHA_162 _comprising the full ectodomain (162 aa) (Figure 1A). Soluble proteins were produced by transfection of 293 T cells and purified from the culture supernatant, as previously described [26]. The molecular size sHA_102 _was approximately 15 kDa (Figure 1B, C), somewhat larger than the predicted size of 11 kDa most likely due to N-linked glycosylation in CRP2 [28]. sHA_162 _migrated predominantly as a 38 kDa species, significantly larger than the predicted 18 kDa for the monomeric form, suggesting incomplete denaturation of the dimeric form that predominates in solution [28].

### Soluble HVEM-mediated HSV-1 infection of CHO-K1 cells

**Figure 2:**
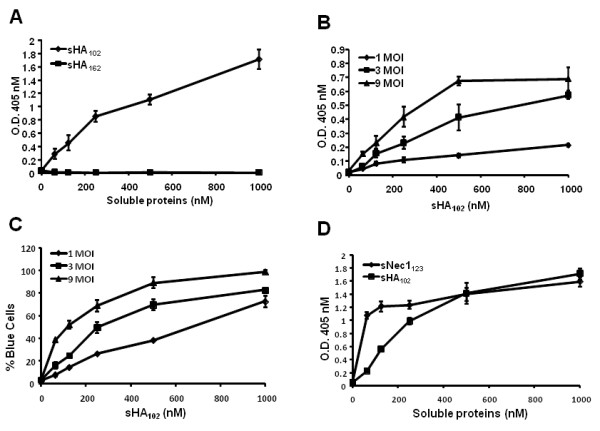
**Soluble HVEM-mediated KOS/tk12 infection of CHO-K1 cells**. Infection was determined by quantitative ONPG assay or X-gal staining. (A) Infection at increasing concentrations of sHA_102 _and sHA_162 _at an MOI of 3. (B) MOI effect on sHA_102_-mediated infection. (C)% infected cells at increasing MOIs determined by X-gal staining of sequential 10-fold dilutions of infected cultures. (D) Comparison of sNec1_123_- and sHA_102_-mediated KOS/tk12 entry (MOI = 3). All values are averages of triplicate determinations.

We determined whether sHA_102 _and sHA_162 _could mediate HSV-1 entry into CHO-K1 cells that are resistant to infection due to the absence of entry receptors. KOS/tk12, expressing LacZ from the viral thymidine kinase (tk) locus in a wild-type (strain KOS) viral background [29], was preincubated at a multiplicity of 3 pfu/cell with CHO-K1 cells in suspension for 1 h at 4°C. Increasing concentrations (0-1,000 nM) of sHA proteins were then added and the cells were incubated for 1 h at 37°C, plated, and incubated for another 7 h at 37°C prior to measurement of β-galactosidase activity by quantitative ONPG assay. As shown in Figure 2A, dose-dependent infection was observed in the presence of sHA_102_, whereas sHA_162 _failed to mediate infection. At an MOI of 9, infection reached a plateau at 500 nM sHA_102 _(Figure 2B), suggesting that either the amount of virus or the number of cells became limiting at this concentration. A similar level of infection was observed at an MOI of 3 with 1,000 nM sHA_102_, arguing for the second interpretation. Indeed, titration of X-gal stained cells showed infection of 90-100% of the cells at an MOI of 9 at 500-1,000 nM sHA_102 _(Figure 2C). These results demonstrated that the known gD binding region of HVEM consisting of CRPs 1 and 2 was sufficient to mediate HSV entry into receptor-deficient cells. As shown in Figure 2D, the efficiency of sHA_102_-mediated entry was comparable to that mediated by the soluble V domain of nectin-1 (sNec_123_) described previously [26].

### Soluble HVEM blocks HSV infection of HVEM-expressing CHO cells

**Figure 4:**
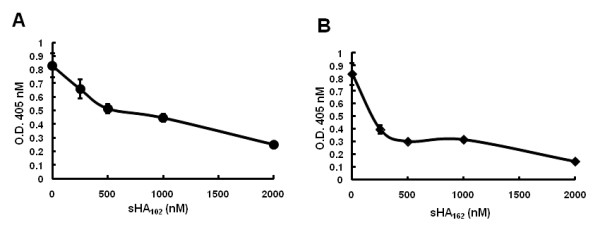
**Inhibition of KOS/tk12 infection of HVEM-expressing CHO cells by soluble HVEM proteins**. Increasing amounts of sHA_102 _(A) or sHA_162 _(B) were preincubated with the virus for 1 h at 4°C prior to infection of CHO-HVEM-12 cells (MOI = 1) for 1 h at 37°C. The cells were washed and infection was measured 7 h later by ONPG assay. Values are averages of triplicate determinations.

Soluble forms of HVEM and nectin-1 have been shown to inhibit HSV-1 entry through the cognate cellular receptors by competition for gD binding [28]. To determine whether the distinct entry-mediating activities of our sHA_102 _and sHA_162 _preparations correlated with their ability to inhibit virus entry via HVEM, we carried out competitive inhibition assays on CHO cells expressing HVEM. Cells were infected with KOS/tk12 in the presence of soluble gD receptors and β-galactosidase expression was measured by ONPG assay. As shown in Figure 4, expression decreased with increasing amounts of sHA_102 _and sHA_162_, indicating dose-dependent entry inhibition. While 1,000 nM of sHA_102 _was required to inhibit KOS/tk12 infection by approximately 50%, sHA_162 _reached this level of inhibition at 250 nM. The greater blocking efficiency of sHA_162 _is in sharp contrast to its lack of entry-mediating activity on receptor-deficient cells. One possible explanation is that CRP 3 and 4, when not anchored in the cell membrane, sterically limit the activation of gD involving partial unfolding to expose its effector/pro-fusion domain [10,12].

### Specific interaction of sHA_102 _with viral gD

**Figure 3:**
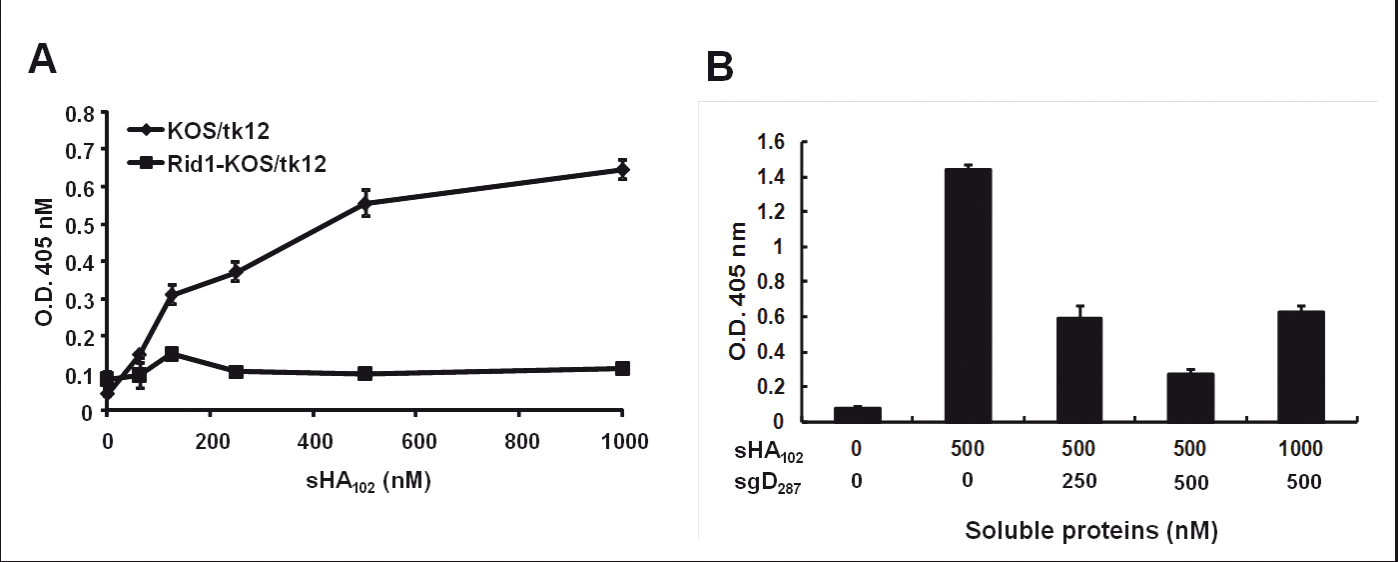
**Specificity of sHA_102_-mediated entry**. (A) Comparison of sHA_102_-mediated CHO-K1 infection by KOS/tk12 and a mutant-gD derivative impaired for HVEM binding, KOS-Rid1/tk12, at MOIs of 3. (B) Inhibition of sHA_102_-mediated infection by soluble gD. sHA_102 _and sgD_287 _at the indicated concentrations were incubated with KOS/tk12 virus at 4°C prior to the addition of CHO-K1 cells (MOI = 3) and incubation at 37°C. Infection was determined by ONPG assay. Values are averages of triplicate determinations.

We determined whether sHA_102_-mediated virus entry into CHO-K1 cells requires specific interaction with gD. As illustrated in Figure 3, while sHA_102 _promoted entry of KOS/tk12 into CHO-K1 cells, little entry was observed by an isogenic mutant virus, KOS-Rid1/tk, that has an amino-acid substitution in gD preventing interaction with HVEM [29]. In addition, we observed that preincubation of sHA_102 _with soluble gD ectodomain (gD_287_; Figure. 1C) inhibited virus entry into CHO-K1 cells in a sgD_287 _dose-dependent manner. When the concentration of sHA_102 _was augmented up to 100 nM, the viral infection was recovered to 2 fold (Figure 3). Together, these results indicated that sHA_102_-mediated HSV entry into CHO-K1 cells occurred through specific binding to viral gD.

### Requirement of heparan sulfate binding for sHA_102_-mediated infection

**Figure 5:**
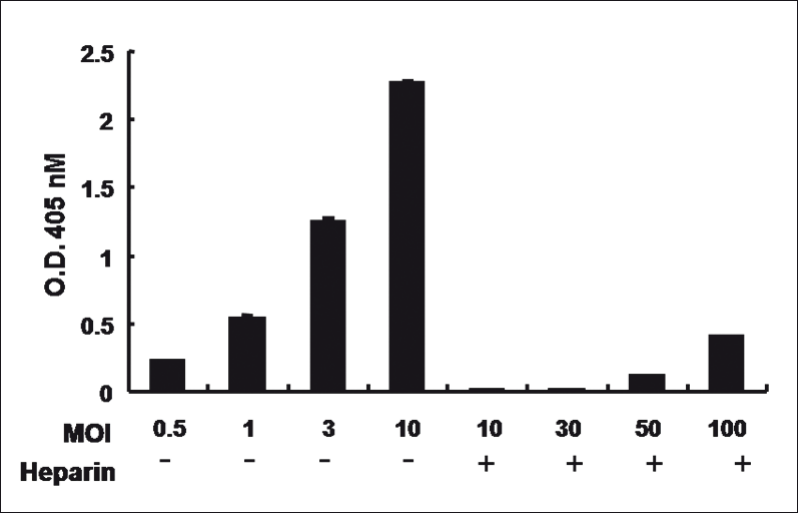
**Heparin inhibition of sHA_102_-mediated KOS/tk12 entry**. Virus and heparin (25 μg/ml) were pre incubated for 30 min at 4°C, CHO-K1 cells were added, and the incubation was continued for another 30 min prior to the addition of sHA_102 _(500 nM), infection as in previous figures, and ONPG assay. Values are averages of triplicate determinations.

HSV attachment is mediated by the binding of gB and gC to cell-surface glycosaminoglycans, particularly heparan sulfate [30]. To determine whether heparan sulfate binding was required for sHA_102_-mediated HSV entry, infections were performed in the presence of 25 μg/ml heparin. As shown in Figure 5, infection was reduced at all MOIs tested, including more than 200-fold at an MOI of 10. These results indicated that virus attachment to cell-surface heparan sulfate is necessary for efficient sHA_102_-mediated HSV entry, as previously observed for soluble nectin-1-mediated infection [26].

### Pathway of sHA_102_-mediated HSV entry into CHO-K1 cells

**Figure 6:**
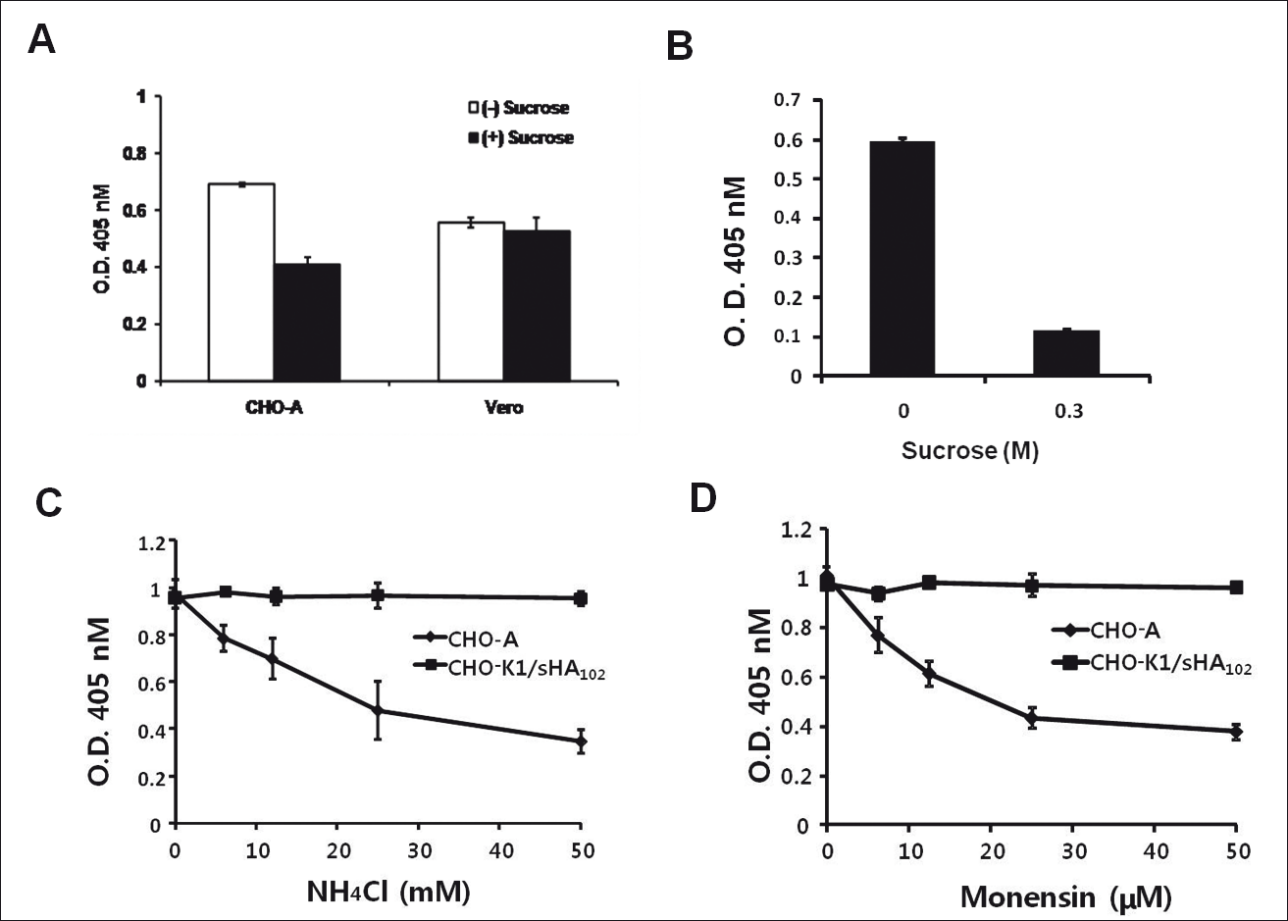
**Effect of agents that inhibit endocytic virion uptake or low pH-dependent infection**. (A) CHO-A and Vero cells were incubated with KOS/tk12 (MOI = 3) in hypertonic medium containing 0.3 M sucrose for 1 h at 4°C and infections were performed in the continuous presence of sucrose followed by ONPG assay. (B) CHO-K1 cells were incubated with KOS/tk12 (MOI = 3) in hypertonic medium containing 0.3 M sucrose for 1 h at 4°C, sHA_102 _was added to 500 nM, and infections were performed in the continued presence of sucrose followed by ONPG assay. (C, D) CHO-K1 cells and CHO-HVEM-12 cells (CHO-A) were pretreated with the indicated amounts of NH_4_Cl (C) or monensin (D) for 30 min at 4°C prior to KOS/tk12 attachment at 4°C (MOI = 3), addition of sHA_102 _to 500 nM (CHO-K1 cells only), and infection as in previous figures in the continued presence of the respective drugs. Infection was measured by ONPG assay. Values are averages of triplicate determinations.

Previous reports have shown that HSV enters into CHO cells expressing human nectin-1 or HVEM via a low pH-dependent endocytic pathway [27]. To examine whether the same entry pathway is used in sHA_102_-induced HSV-1 infection of CHO-K1 cells, we treated the cells with hypertonic medium or lysosomotropic agents. Medium containing 0.3 M sucrose reduced sHA_102_-mediated KOS/tk12 infection approximately 5-fold (Figure 6B), similar to results reported for HSV infection of HVEM-expressing CHO cells (Figure 6A) [27], indicating an important role for active endocytosis in entry mediated by sHA_102_. To determine whether endosomal acidification was required for infection, CHO-K1 cells were pre-treated with increasing concentrations of NH_4_Cl or monensin, and incubated with KOS/tk12 and sHA_102 _in the continued presence of these agents. The results showed no inhibition of sHA_102_-mediated infection while infection of CHO-HVEM cells was sensitive to these agents (Figure 6C, D). Thus, unlike HSV infection of HVEM-expressing CHO cells, sHA_102_-mediated CHO-K1 infection appears to take place by a low pH-independent endocytic mechanism.

## Discussion

In this study, we demonstrate that a soluble gD receptor consisting of the N-terminal 102 amino acids of HVEM is an efficient mediator of HSV-1 infection of receptor-negative, HSV-resistant CHO-K1 cells. In contrast, sHA_162_, comprising the complete HVEM ectodomain, did not enable viral entry although both sHA_102 _and sHA_162 _blocked virus infection of receptor-bearing CHO cells. We suggest that sHA_162 _binds to viral gD but prevents the folding of gD into an active conformation that can trigger the fusion process. Using inhibitors of receptor- and pH-dependent endocytosis, we obtained evidence that sHA_102_-mediated infection of CHO-K1 cells occurs by pH-independent endocytosis, unlike the low pH-dependent endocytic mechanism responsible for infection of CHO-HVEM cells [27].

Previous studies have reported inhibition of HSV infection of CHO-HVEM cells by the soluble ectodomain of HVEM, alone [HVEM (200 t)] or as a fusion with the Fc region of rabbit immunoglobulin G (IgG) heavy chain [6,28]. In a later study, Whitbeck and colleagues also reported the ability of HVEM (200 t) to promote HSV entry into receptor-deficient CHO-K1 cells [31]. Although our sHA_162 _had essentially the same primary sequence as HVEM (200 t), we did not observe an entry-promoting effect of sHA_162 _on CHO-K1 cells. A potentially significant difference with our study is that Whitbeck et al. [31] used a low speed centrifugation technique referred to as spinoculation to augment HVEM(200 t)-mediated virus infection. Spinoculation has been used with HSV and other enveloped viruses to bring virion and cell membranes closely together [21,32-34], for example to bypass receptor-dependent virus attachment in studies demonstrating soluble receptor-mediated infection by avian sarcoma and leukosis viruses [35,36]. Since HSV attachment to cells is mediated mainly by viral envelope proteins other than gD, we performed our experiments without spinoculation while showing that heparin-sensitive virus attachment was essential under these conditions. We observed highly efficient sHA_102_-mediated infection (> 80% infection at an MOI of 3), but entry mediated by the complete soluble ectodomain was essentially undetectable, suggesting that spinoculation in the study by Whitbeck et al. [31] had allowed the detection of HVEM (200 t) activity by concentrating the virus at the cell surface to raise the sensitivity of the assay. Nonetheless, the Whitbeck study reported a maximum HVEM (200 t)-mediated increase in infection of CHO-K1 cells of 15.5-fold [5 μM HVEM (200 t); MOI = 2], substantially less than the increases we observed at lower sHA_102 _concentrations (Figure 2). Thus, while sHA_162_/HVEM (200 t) may not be inactive in infection of receptor-deficient cells, sHA_102 _is clearly more effective.

The similar inhibition of CHO-HVEM infection by sHA_102 _and sHA_162 _indicated that the difference in entry-mediating activity between these two proteins is not due to differences in gD binding, suggesting that the extra sequences in sHA_162_/HVEM(200 t) compared to sHA_102 _negatively affect the efficiency of gD activation. The previously reported crystal structure of the gD ectodomain complexed with the HVEM ectodomain has shown that the N-terminal region of gD undergoes a conformational change during HVEM binding [8]. It is conceivable that sHA_102 _either induces a more global change in the gD conformation or that the C-terminal portion of sHA_162_/HVEM(200 t) interferes with the efficient receptor-dependent unmasking of the gD effector/pro-fusion domain proposed by Krummenacher et al. [12] and Fusco et al. [10].

In the absence of gD receptors, HSV is internalized by CHO cells but fails to enter the cytoplasm and is degraded in lysosomes [37]. The presence of gD receptors allows pH-dependent viral capsid escape from endosomes and release into the cytosol [37]. Whitbeck et al. [31] have described evidence that HVEM (200 t) "primes" gD for activation of the membrane fusion machinery, but that a mildly acidic pH is required in CHO cells as a second signal for fusion initiation. Surprisingly, our results using NH_4_Cl or monensin to neutralize the endosomal pH did not show a reduction in sHA_102_-mediated infection although virus uptake by endocytosis was indicated by reduced infection in media containing 0.3 M sucrose. Thus it appears that the second signal is not required for sHA_102 _activation of gD. At the opposite end, it appears that endosome acidification as the second signal is not sufficient for efficient activation of gD by sHA_162_/HVEM (200 t). Thus our results suggest that both situations differ from normal virus entry via full-length HVEM. It is of interest that several examples have been reported of cell-surface HVEM failing to serve as a functional HSV entry receptor [38-40], raising the possibility that these cells lack a mechanism to counteract interference by CRPs 3 and 4 with gD activation by full-length HVEM. Our study suggests that sHA_102 _may be an efficient tool to enable HSV vector-mediated delivery of therapeutic genes to receptor-deficient cells.

## Conclusions

Here we have demonstrated a soluble gD receptor, containing N-terminal 102 amino acids of HVEM, for efficient HSV infection into HSV receptor-negative, HSV-resistant CHO-K1 cells. This study is consistent with two other soluble form of gD receptors, sNec1_123 _and soluble 3-*O*S HS. We can improve the usage of sHA_102 _by fusing it with ligands or receptors of disease related antigens as a promising molecule for retargeting of natural tropism of HSV-1. This retargeting strategy can be combined to create specific recognition by genetic modifications of the virion envelope for incorporation of targeting moiety.

## Methods

### Cells and viruses

African green monkey kidney (Vero) cells were obtained from American Type Culture Collection (ATCC, Rockville, MD) and maintained in Dulbecco's modification of Eagle minimal medium (DMEM) supplemented with 10% fetal bovine serum (FBS). Chinese hamster ovary cells (CHO-K1, ATCC) were maintained in Ham's F-12 K medium with 10% FBS, 100 U/ml penicillin, and 100 ug/ml streptomycin (Invitrogen, Carlsbad, CA). CHO-HVEM-12 cells (Montgomery et al., 1996) are CHO-K1 derivatives constitutively expressing full-length human HVEM and were kindly provided by Patricia G. Spear (Northwestern University, IL, USA). Both cell lines were grown in Ham's F-12 K medium supplemented with 10% FBS and 400 μg/ml G418 (Invitrogen).

Wild-type and mutant gD β-galactosidase reporter viruses KOS/tk12 and KOS-Rid1/tk12 [29] were kindly provided by Patricia G. Spear and were propagated and titered on Vero cells.

### Soluble protein expression constructs

Sequences encoding sHA_102 _and sHA_162 _were amplified by PCR on HVEM plasmid pBEC14 ([6]; a kind gift from Patricia Spear) using the following primers: sHA_102_/sHA_162 _sense primer, 5'-CCC AAG CTT GCC ACC ATG GAG CCT CCT GGA GAC TGG GGG CC-3'; sHA_102 _anti-sense primer, 5'-CCC CTC GAG CTA ATG GTG ATG GTG ATG GTG AGC GCG GCA CGC GGC GCA-3'; sHA_162 _anti-sense primer, 5'-CCC CTC GAG CTA GTG GTG GTG GTG GTG GTG GGA GCT GCT GGT CCC AGC-3'. Primers were designed to provide a Kozak translational initiation sequence at the start of the open reading frame (ORF) and six histidine residues followed by a TAG stop codon at the end. Underlined sequences indicate recognition sites for the restriction enzymes *Hind *III and *Xho *I. Following digestion with *Hind *III plus *Xho *I, each PCR product was cloned into pcDNA3.1 (+) (Invitrogen), yielding mammalian expression constructs pHA_102 _and pHA_162_.

Expression plasmids for sNec1_123 _and sgD_287 _were as previously described [26].

### Production and purification of His fusion proteins

Soluble proteins were expressed in 293 T cells and purified using the Probond™ resin purification system (Invitrogen) according to the manufacturer's protocol. Briefly, DNA transfections were performed with LipofectAmine Plus reagent (Invitrogen) and the cells were then cultured in serum-free media. Culture supernatants were harvested at 72 h post-transfection, mixed with Probond resin, and incubated for 1 hr at 4°C. The resin was washed and bound proteins were eluted.

### SDS-PAGE and Western blotting

Purified soluble proteins were electrophoresed on a 12% polyacrylamide-sodium dodecyl sulfate (SDS) gel and blotted onto Protran nitrocellulose membrane (Whatman GmbH, Dassel, Germany). The membrane was blocked with a solution of 3% skim milk in PBS/0.1% Tween-20, and incubated for 1 h at room temperature with anti-His antibody (Santa Cruz Biotechnology, Santa Cruz, CA) diluted in blocking buffer. The membrane was washed three times and treated with horseradish peroxide-conjugated goat anti-mouse IgG (Sigma, St. Louis, MO) for 1 h at room temperature. Peroxidase activity was detected using an ECL kit (Amersham Pharmacia Biotech, Piscataway, NJ).

### HSV entry assay

CHO-K1 cells in suspension (6 × 10^4 ^cells/well) were preincubated with KOS/tk12 virus in PBS in a total volume of 40 μl for 1 h at 4°C on a rocking device. Increasing concentrations of soluble proteins (0 - 1,000 nM) were added to the virus/cell mixture and incubated for 1 h at 37°C. The cells were collected by centrifugation, washed twice with PBS, resuspended in 40 μl of Ham's F-12 K medium, and plated in a single well of a 96 well plate. After continued incubation at 37°C for 7 h, virus entry was detected by staining with X-gal (5-bromo-4-chloro-3-indolyl-β-D-galactopyranoside) or by colorimetric assay (ONPG, *o*-nitrophenyl-β-D-galactopyranoside), as previously described [41]. Briefly, infected cell monolayers were fixed with 0.2% glutaldehyde (Sigma) for 15 min at room temperature (RT), washed with PBS, and incubated with 0.2 mg/ml X-gal (Sigma);% infection was determined by staining of sequential 10-fold dilutions of infected cells. For the colorimetric assay, infected monolayers were washed with PBS and lysed in 150 μl of 1% NP-40, 1 mM MgCl_2_, 50 mM β-mercaptoethanol, and 4 mg/ml ONPG (Sigma). Lysates were incubated at 37°C until a yellow color developed, and absorbance was measured at 405 nM.

### Infection inhibition by soluble receptors

Different concentrations of soluble receptors (0-2,000 nM) were preincubated with 6 × 10^4 ^pfu of KOS/tk12 in a total volume of 40 μl for 1 h at 4°C. CHO-HVEM-12 cells (6 × 10^4 ^cells/well) were added and the mixture was incubated for 1 h at 37°C. The cells were then collected, washed twice with PBS, resuspended in 40 μl of Ham's F-12 K medium, and incubated for 7 h at 37°C. Infection was measured by colorimetric ONPG assay, as described above.

### Infection inhibition by sgD_287_

Experiments to determine the sensitivity of sHA_102_-mediated infection to competition for sHA_102 _binding with soluble gD were performed essentially as described previously for sNec1_123 _(Kwon et al., 2006). Briefly, various amounts of sHA_102 _and sgD_287 _were incubated with KOS/tk12 in PBS for 1 h at 4°C on rocking device. A total of 5 × 10^5 ^CHO-K1 cells (MOI = 3) were added and the mixture was incubated for 1 h at 37°C. The cells were collected, washed, plated with complete medium, and incubated for another 7 h. Infection was measured by ONPG assay.

### Heparin inhibition

Increasing amounts of KOS/tk12 were preincubated with 25 μl/ml heparin for 30 min at 4°C, CHO-K1 cells were added, and the incubation was continued for another 30 min. sHA_102 _to a final concentration of 500 nM was then added and the mixture was incubated for 1 h at 37°C. The cells were washed, plated, incubated with complete medium for 7 h at 37°C, and infection was determined by ONPG assay.

### Endocytosis inhibition assays

KOS/tk12 (MOI = 3) was preincubated with CHO-K1 cells for 30 min at 4°C in the presence of 0.3 M sucrose, sHA_102 _(500 nM final concentration) was added, and the mixture was incubated for 1 h at 37°C. Alternatively, CHO-K1 cells were incubated with different concentrations of ammonium chloride (NH_4_Cl) or monensin (Sigma) for 30 min at 4°C, virus was added and the incubation continued for another 30 min at 4°C, and sHA_102 _was added with incubation for 1 h at 37°C. In each situation, the cells were washed, plated, and incubated for 7 h at 37°C in complete medium containing the respective inhibitory agents. Virus entry was measured by ONPG assay.

### Statistical analysis

All results were performed in triplicate and the values are expressed as means ± SD. P values were calculated by using Student's *t *test, and statistical significance was defined as *P *≤ 0.05.

## Abbreviations

BTLA: B- and T-lymphocyte attenuator; CRPs: Cysteine-rich pseudo repeats; FBS: Fetal bovine serum; gC: Glycoproteins C; gD: Glycoprotein D; HVEM or HveA: Herpes virus entry mediator; HSV-1: Herpes simplex virus type 1; Lt-α: Lymphotoxin alpha; ONPG: o-nitrophenyl-β-D-galactopyranoside; ORF: Open reading frame; PILRα: Paired immunoglobulin-like type-2 receptor; Tk: Thymidine kinase; TNFR: Tumor necrosis factor receptor; X-gal: 5-bromo-4-chloro-3-indolyl-β-D-galactopyranoside; 3-OS HS: 3-*O*-sulfated heparan sulfate.

## Competing interests

The authors declare that they have no competing interests.

## Authors' contributions

HB and HK designed the research; HB and YN performed research; JK analyzed data; HB and HK contributed to drafting the manuscript. All authors read and approved the final manuscript.

## References

[B1] Herold BC Visalli RJ Susmarski N Brandt CR Spear PG Glycoprotein C-independent binding of herpes simplex virus to cells requires cell surface heparan sulphate and glycoprotein B J Gen Virol 1994 75 1211 1222 10.1099/0022-1317-75-6-1211 8207388

[B2] Laquerre S Argnani R Anderson DB Zucchini S Manservigi R Glorioso JC Heparan sulfate proteoglycan binding by herpes simplex virus type 1 glycoprotein B and C, which differ in their contribution to virus attachment, penetration, and cell-to-cell spread J Virol 1998 72 6119 6130 9621076 10.1128/jvi.72.7.6119-6130.1998 PMC110418

[B3] Lycke E Johansson M Svennerholm B Lindahl U Binding of herpes simplex virus to cellular heparan sulphate, an initial step in the adsorption process J Gen Virol 1991 72 1131 1137 10.1099/0022-1317-72-5-1131 1851813

[B4] Banfield BW Leduc Y Esford L Schubert K Tufaro F Sequential isolation of proteoglycan synthesis mutants by using herpes simplex virus as a selective agent: evidence for a proteoglycan-independent virus entry pathway J Virol 1995 69 3290 3298 7745676 10.1128/jvi.69.6.3290-3298.1995 PMC189040

[B5] Cocchi F Menotti L Mirandola P Lopez M Campadelli-Fiume G The ectodomain of a novel member of the immunoglobulin subfamily related to the poliovirus receptor has the attributes of a bona fide receptor for herpes simplex virus types 1 and 2 in human cells J Virol 1998 72 9992 10002 9811737 10.1128/jvi.72.12.9992-10002.1998 PMC110516

[B6] Montgomery R Warner MS Lum BJ Spear PG Herpes simplex virus-1 entry into cells mediated by a novel member of the TNF/NGF receptor family Cell 1996 87 427 436 10.1016/S0092-8674(00)81363-X 8898196

[B7] Shukla D Liu J Blaiklock P Shworak NW Bai X Esko JD Cohen GH Eisenberg RJ Rosenberg RD Spear PG A novel role for 3-*O*-sulfated heparan sulfate in herpes simplex virus 1 entry Cell 1999 99 13 22 10.1016/S0092-8674(00)80058-6 10520990

[B8] Carfi A Willis SH Whitbeck JC Krummenacher C Cohen GH Eisenberg RJ Willey DC Herpes simplex virus glycoprotein D bound to the human receptor HveA Mol Cell 2001 8 169 179 10.1016/S1097-2765(01)00298-2 11511370

[B9] Campadelli-Fiume G Amasio M Avitabile E Cerretani A Forghieri C Gianni T Menotti L The multipartite system that mediates entry of herpes simplex virus into the cell Rev Med Virol 2007 17 313 326 10.1002/rmv.546 17573668

[B10] Fusco D Forghieri C Campadelli-Fiume G The pro-fusion domain of herpes simplex virus glycoprotein D (gD) interacts with the gD M terminus and is displaced by soluble forms of viral receptors Proc Natl Acad Sci USA 2005 28 9323 9328 10.1073/pnas.0503907102 PMC1166633 15972328

[B11] Gianni T Amasio M Campadelli-Fiume G Herpes simplex virus gD forms distinct complexes with fusion executors gB and gH/L in part through the C-terminal profusion domain J Biol Chem 2009 284 17370 17382 10.1074/jbc.M109.005728 19386594 PMC2719377

[B12] Krummenacher C Supekar VM Whitbeck JC Lazear E Connolly SA Eisenberg RJ Cohen GH Wiley DC Carfi A Structure of unliganded HSV gD reveals a mechanism for receptor-mediated activation of virus entry EMBO J 2005 24 4144 4153 10.1038/sj.emboj.7600875 16292345 PMC1356314

[B13] Satoh T Arii J Suenaga T Wang J Kogure A Uehori J Arase N Shiratori I Tanaka S Kawaguchi Y Spear PG Lanier LL Arase H PILRalpha is a herpes simplex virus-1 entry coreceptor that associates with glycoprotein B Cell 2008 132 935 944 10.1016/j.cell.2008.01.043 18358807 PMC2394663

[B14] Arii J Goto H Suenaga T Oyama M Kozuka-Hata H Imai T Minowa A Akashi H Arase H Kawaoka Y Kawaguchi Y 2010. Non-muscle myosin IIA is a functional entry receptor for herpes simplex virus-1 Nature 2010 467 859 862 10.1038/nature09420 20944748

[B15] Geraghty RJ Krummenacher C Cohen GH Eisenberg RJ Spear PG Entry of alphaherpesviruses mediated by poliovirus receptor-related protein 1 and poliovirus receptor Science 1998 280 1618 1620 10.1126/science.280.5369.1618 9616127

[B16] Takai Y Nakanishi H Nectin and afadin: novel organizers of intercellular junctions J Cell Sci 2003 116 17 27 10.1242/jcs.00167 12456712

[B17] Krummenacher C Rux AH Whitbeck JC Pronce-de-Leon M Lou H Baribaud I Hou W Zou C Geraghty RJ Spear PG Eisenberg RJ Cohen GH The first immunoglobulin-like domain of HveC is sufficient to bind herpes simplex virus gD with full affinity, while the third domain is involved in oligomerization of HveC J Virol 1999 73 8127 8137 10482562 10.1128/jvi.73.10.8127-8137.1999 PMC112829

[B18] Spear PG Herpes simplex virus: receptors and ligands for cell entry Cell Microbiol 2004 6 401 410 10.1111/j.1462-5822.2004.00389.x 15056211

[B19] Kwon BS Tan KB Ni J Oh KO Lee ZH Kim KK Kim YJ Wang S Gentz R Yu GL Harrop J Lyn SD Silverman C Porter TG A newly identified member of the tumor necrosis factor receptor superfamily with a wide tissue distribution and involvement in lymphocyte activation J Biol Chem 1997 272 14272 14276 10.1074/jbc.272.22.14272 9162061

[B20] Cai G Freeman GJ The CD160, BTLA, LIGHT/HVEM pathway: a bidirectional switch regulating T-cell activation Immunol Rev 2009 229 244 258 10.1111/j.1600-065X.2009.00783.x 19426226

[B21] O'Doherty U Swiggard WJ Malim MH Human immunodeficiency virus type 1 spinoculation enhances infection through virus binding J virol 2000 74 10074 10080 10.1128/JVI.74.21.10074-10080.2000 11024136 PMC102046

[B22] Dean HJ Terhune SS Shieh MT Susmarski N Spear PG Single amino acid substitutions in gD of herpes simplex virus 1 confer resistance to gD-mediated interference and cause cell-type-dependent alterations in infectivity Virology 1994 199 67 80 10.1006/viro.1994.1098 8116256

[B23] Connolly SA Landsburg DJ Carfi A Willey DC Cohen GH Eisenberg RJ Structure-based mutagenesis of herpes simplex virus glycoprotein D defines three critical regions at the gD-HveA/HVEM binding interface J Virol 2003 77 8127 8140 10.1128/JVI.77.14.8127-8140.2003 12829851 PMC161942

[B24] Yoon M Zago A Shukla D Spear PG Mutations in the N termini of herpes simplex virus type 1 and 2 gDs alter functional interactions with the entry/fusion receptors HVEM, nectin-2, and 3-*O*-sulfated heparan sulfate but not with nectin-1 J Virol 2003 77 9221 9231 10.1128/JVI.77.17.9221-9231.2003 12915538 PMC187404

[B25] Whitbeck JC Connolly SA Willis SH Hou W Krummenacher C Ponce de Leon M Lou H Baribaud I Eisenberg RJ Cohen GH Localization of the gD-binding region of the human herpes simplex virus receptor, HveA J Virol 2001 75 171 180 10.1128/JVI.75.1.171-180.2001 11119586 PMC113910

[B26] Kwon H Bai Q Baek HJ Felmet K Burton EA Goins WF Cohen JB Glorioso JC Soluble V domain of nectin-1/HveC enables entry of herpes simplex virus type 1 (HSV-1) into HSV-resistant cells by binding to viral glycoprotein D J Virol 2006 80 138 148 10.1128/JVI.80.1.138-148.2006 16352538 PMC1317534

[B27] Nicola AV McEvoy AM Straus SE Roles for endocytosis and low pH in herpes simplex virus entry into HeLa and Chinese hamster ovary cells J Virol 2003 77 5324 5332 10.1128/JVI.77.9.5324-5332.2003 12692234 PMC153978

[B28] Whitbeck JC Peng C Lou H Xu R Willis SH Ponce de Leon M Peng T Nicola AV Montgomery RI Warner MS Soulika AM Spruce LA Moore WT Lambris JD Spear PG Cohen GH Eisenberg RJ Glycoprotein D of herpes simplex virus (HSV) binds directly to HVEM, a member of the tumor necrosis factor receptor superfamily and a mediator of HSV entry J Virol 1997 71 6083 6093 9223502 10.1128/jvi.71.8.6083-6093.1997 PMC191868

[B29] Warner MS Geraghty RJ Martinez WM Montgomery RI Whitbeck JC Xu R Eisenberg RJ Cohen GH Spear PG A cell surface protein with herpesvirus entry activity (HveB) confers susceptibility to infection by mutants of herpes simplex virus type 1, herpes simplex virus type 2, and pseudorabies virus Virology 1998 246 179 189 10.1006/viro.1998.9218 9657005

[B30] O'Donnell CD Shukla The importance of heparan sulfate in herpesvirus infection Virol Sin 2008 23 383 393 10.1007/s12250-008-2992-1 19956628 PMC2778322

[B31] Whitbeck JC Zuo Y Milne RS Cohen GH Eisenberg RJ Stable association of herpes simplex virus with target membranes is triggered by low pH in the presence of the gD receptor, HVEM J Virol 2006 80 3773 3780 10.1128/JVI.80.8.3773-3780.2006 16571794 PMC1440471

[B32] Hudson JB Further studies on the mechanism of centrifugal enhancement of cytomegalovirus infectivity J Virol Methods 1998 19 97 108 10.1016/0166-0934(88)90153-x 2835385

[B33] Scanlan PM Tiwari V Bommireddy S Shukla D Spinoculation of heparan sulfate deficient cells enhances HSV-1 entry, but does not abolish the need for essential glycoproteins in viral fusion J Virol Methods 2005 128 104 112 10.1016/j.jviromet.2005.04.008 15908019

[B34] Tenser RB Dunstan ME Mechanisms of herpes simplex virus infectivity enhanced by ultracentrifugal inoculation Infect Immun 1980 30 193 197 6254878 10.1128/iai.30.1.193-197.1980 PMC551294

[B35] Damico R Bates P Soluble receptor-induced retroviral infection of receptor-deficient cells J Virol 2000 74 6469 6475 10.1128/JVI.74.14.6469-6475.2000 10864659 PMC112155

[B36] Knauss DJ Young JA A fifteen-amino-acid TVB peptide serves as a minimal soluble receptor for subgroup B avian leukosis and sarcoma viruses J Virol 2002 76 5404 5410 10.1128/JVI.76.11.5404-5410.2002 11991969 PMC137033

[B37] Nicola AV Straus SE Cellular and viral requirements for rapid endocytic entry of herpes simplex virus J Virol 2004 78 7508 7517 10.1128/JVI.78.14.7508-7517.2004 15220424 PMC434080

[B38] Manoj S Jogger CR Myscofski D Yoon M Spear PG Mutations in herpes simplex virus glycoprotein D that prevent cell entry via nectins and alter cell tropism Proc Natl Acad Sci USA 2004 101 12414 12421 10.1073/pnas.0404211101 15273289 PMC515077

[B39] Uchida H Shah WA Ozuer A Frampton AR Jr Goins WF Grandi P Cohen JB Glorioso JC Generation of herpesvirus entry mediator (HVEM)-restricted herpes simplex virus type 1 mutant viruses: resistance of HVEM-expressing cells and identification of mutations that rescue nectin-1 recognition J Virol 2009 83 2951 2961 10.1128/JVI.01449-08 19129446 PMC2655597

[B40] Tiwari V Oh MJ Kovacs M Shukla SY Valyl-Nagy T Shukla D Role for nectin-1 in herpes simplex virus 1 entry and spread in human retinal pigment epithelial cells FEBS J 2008 275 5272 5285 10.1111/j.1742-4658.2008.06655.x 18803666 PMC2758088

[B41] Nakano K Asano R Tsumoto K Kwon H Goins WF Kumagai I Cohen JB Glorioso JC Herpes simplex virus targeting to the EGF receptor by a gD-specific soluble bridging molecule Mol Ther 2005 11 617 626 10.1016/j.ymthe.2004.12.012 15771964

